# Caseous calcification of the mitral annulus: computed tomography features

**DOI:** 10.1590/0100-3984.2015.0096

**Published:** 2016

**Authors:** Fernanda Boldrini Assunção, Diogo Costa Leandro de Oliveira, Alair Augusto Sarmet Moreira Damas dos Santos, Marcelo Souto Nacif

**Affiliations:** 1 Complexo Hospitalar de Niterói (CHN), Niterói, RJ, Brazil.; 2 Universidade Federal Fluminense (UFF), Niterói, RJ, Brazil.

**Figure 1:**
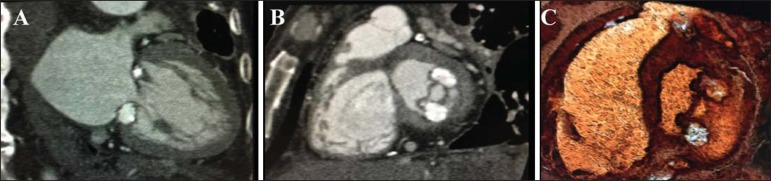
Degenerative caseous calcification of the mitral valve. **A:** Long axis two chambers showing coarse caseous calcifications between the anterior and posterior commissures. **B:** Short axis (in the mitral valve plane) showing caseous calcifications, together with significant restriction of the mitral valve orifice. **C:** Volume rendering reconstruction confirming the diagnosis of degenerative caseous calcification of the mitral annulus.

*Dear Editor*,

A 62-year-old patient with chronic kidney disease, who was undergoing treatment with intermittent dialysis, was admitted to the hospital for investigation of a complaint of progressively worsening dyspnea, despite the optimization of the dialysis. To elucidate the case, ancillary tests were ordered, such tests including echocardiography. The echocardiography showed an expansive formation in the mitral valve, and cardiac computed tomography (CCT) was performed in order to better evaluate that finding (Figure 1). The CCT identified a coarse caseous calcification between the anterior and posterior commissures, accompanied by a significant reduction in the size of the mitral valve orifice, with a maximum aperture of 0.7 cm^3^, as determined by planimetry. The CCT images allowed the diagnosis of degenerative caseous calcification of the mitral annulus.

Improving the use of imaging methods in the evaluation of cardiovascular diseases has been the objective of a number of recent studies in the radiology literature of Brazil^(1-5)^. Caseous calcification of the mitral annulus is a chronic degenerative process that usually involves the posterior mitral annulus^(6)^. It is most prevalent in elderly females^(7)^ and in patients with chronic kidney disease who are on hemodialysis^(8-10)^. It is a rare disease, accounting for only 0.5-1.0% of all calcifications of the mitral annulus. Although rare, it is one of the major differential diagnoses of cardiac tumors, thrombi, vegetations, and abscesses^(11)^.

In most cases are asymptomatic patients, and the diagnosis is established by examination of cardiac imaging performed for other purposes. The symptoms, when present, correspond to palpitations, dyspnea, and syncope^(11)^. The prognosis of caseous degeneration of the mitral annulus is good, especially in patients who are asymptomatic, although some patients develop severe symptomatic valvular dysfunction; in the latter group of patients, the prognosis is poor and surgery should be considered^(9,12)^.

On the CCT scans, we noted a hyperintense crescent-shaped mass or a well-defined oval-shaped mass with peripheral calcification, usually along the posterior mitral annulus, which was not enhanced after contrast administration^(13)^. The heterogeneity of the content of the mass was confirmed by the variation in its density, which can range from negative Hounsfield units, suggesting fatty degeneration, to elevated Hounsfield units, suggesting a high protein content and structural calcification^(14)^. The central hypointensity was secondary to liquefaction of the calcium that fills the center of mass^(11,13,15)^.

In this context of our findings in the case presented here, we can conclude that CCT helps confirm the diagnosis, allows the degree of mitral valve stenosis to be evaluated, and offers measures to improve treatment strategies, especially those involving transcatheter or percutaneous transapical mitral valve implantation. Therefore, CCT is considered an excellent tool for the diagnosis of caseous degeneration of the mitral annulus.
